# Red Cell Pyruvate Kinase Deficiency With Hypertriglyceridemia: A Case Report

**DOI:** 10.7759/cureus.65839

**Published:** 2024-07-31

**Authors:** Dinesh V Hinge, Mamta Muranjan, Amar Taksande, Priyanka Hampe

**Affiliations:** 1 Pediatrics and Child Health, Jawaharlal Nehru Medical College, Datta Meghe Institute of Higher Education and Research, Wardha, IND; 2 Pediatrics and Child Health, Seth Gordhandas Sunderdas Medical College (GSMC) and the King Edward Memorial (KEM) Hospital, Mumbai, IND

**Keywords:** hypertriglyceridemia, milky blood sample, regular blood transfusion, hemolytic anemia, pyruvate kinase deficiency

## Abstract

Red cell pyruvate kinase (PK) deficiency is a genetic disorder affecting the enzyme PK in red blood cells. A deficiency in PK leads to hemolytic anemia. Hypertriglyceridemia means elevated levels of triglycerides in the blood. The hypertriglyceridemia disorder can be primary or secondary to an underlying disease. Hypertriglyceridemia with β-thalassemia major is a known association and is called hypertriglyceridemia-thalassemia syndrome. A four-month-old male child was found to have milky serum. On investigation, there was severe anemia, with triglycerides at 1197 mg/dL and high lactate dehydrogenase (LDH). The child had severe pallor, mild icterus, a dysmorphic face, and splenohepatomegaly. Ophthalmic examination showed lipemia retinitis. The child was treated with medium-chain fatty acid formula feed. Regular blood transfusions, folic acid supplements, and avoidance of salicylate group drugs were advised. The child improved and is doing well. Thus, early diagnosis and treatment can change the prognosis and help maintain a near-normal life for affected infants.

## Introduction

Red cell pyruvate kinase (PK) deficiency is the most predominant glycolytic pathway abnormality that causes hereditary non-spherocytic hemolytic anemia. The clinical manifestation varies, ranging from minor symptoms to critically ill newborn anemia and jaundice that need to be treated in intensive care [1]. It is an autosomal recessive condition caused by a PK-LR gene mutation [1]. The presentation will be on severe neonatal anemia, chronic hemolysis, and neonatal jaundice [2]. Treatment of this disease is primarily supportive: blood transfusion, splenectomy, and definitive treatment like bone marrow transplant [2]. Acute or chronic hemolysis is associated with hypertriglyceridemia, which can be acquired or hereditary and exhibits enhanced synthesis or reduced catabolism [3]. Hypertriglyceridemia can be primary or secondary. Primary familial hypertriglyceridemia is autosomal dominant. Secondary hemolysis causes transient hyperlipidemia through direct red cell destruction or intravascular coagulation [4]. The association between hypertriglyceridemia and red cell PK deficiency is seen in the medical literature.

## Case presentation

**Figure 1:**
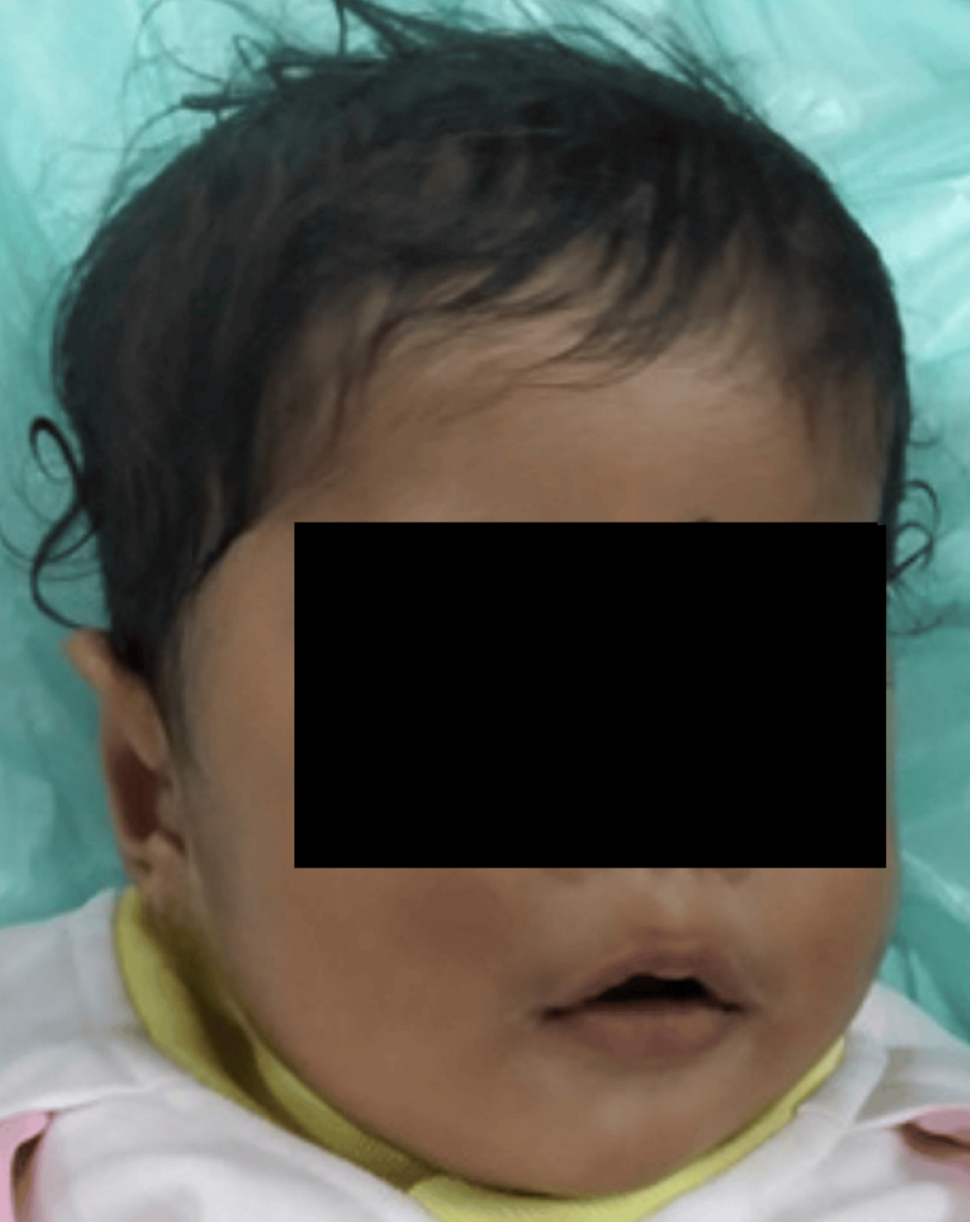
Dysmorphic face and frontal bossing

A four-month-old male child, born of third-degree consanguinity, presented with pallor since birth and abdominal distension for the past two months. The baby was born by normal vaginal delivery. After 12 hours of life, the baby developed severe respiratory distress and indirect hyperbilirubinemia. Respiratory support was provided, and an exchange transfusion was performed for neonatal hyperbilirubinemia, followed by two days of phototherapy. The child has a history of two hospitalizations for low hemoglobin, during which packed red blood cell transfusions were administered. On head-to-toe examination, the child exhibited severe pallor and mild icterus. Additionally, dysmorphic facial features and frontal bossing were observed, as shown in Figure 1.

**Figure 2:**
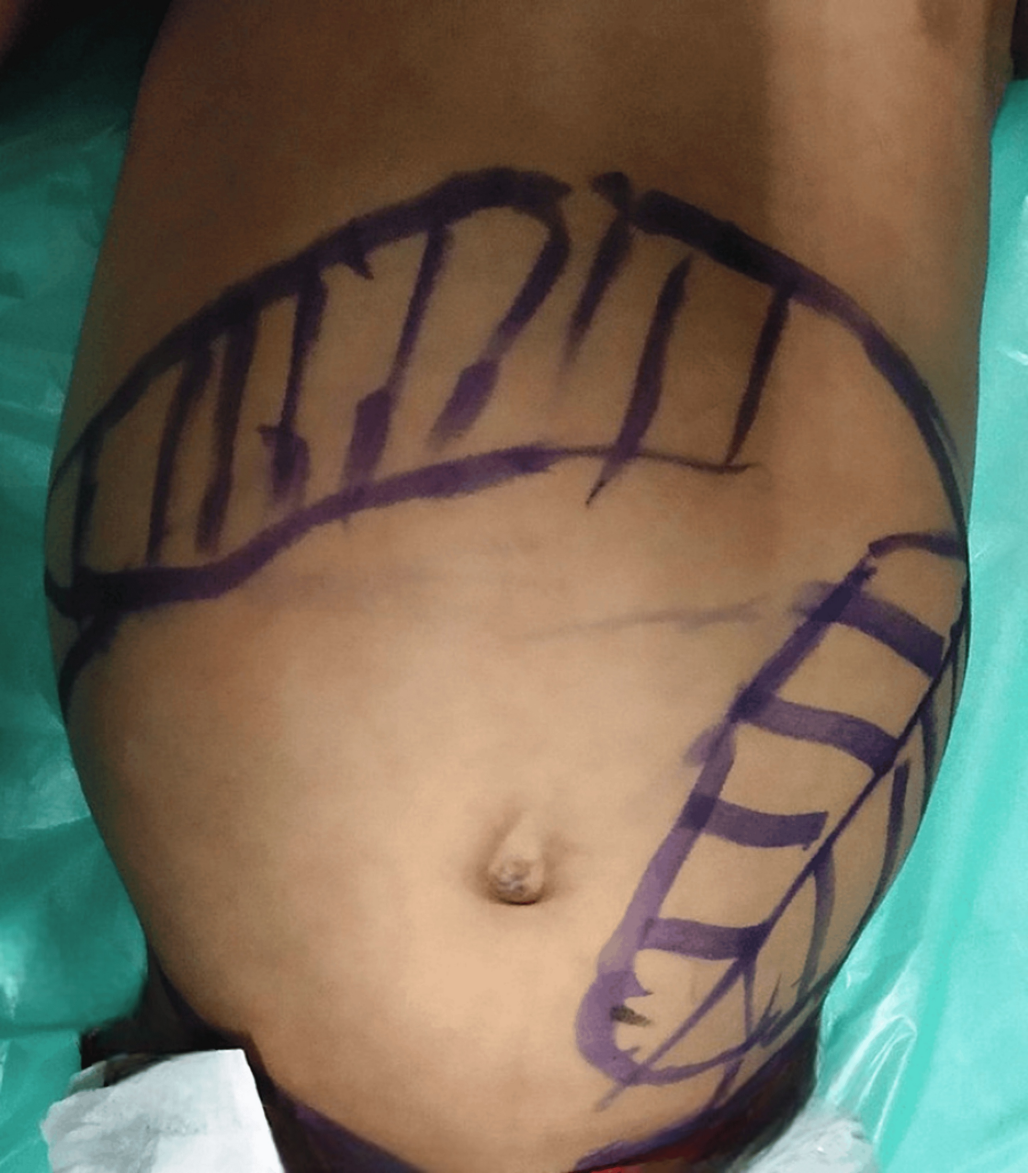
Enlarged spleen and liver size (splenohepatomegaly)

Weight and height were on the third centile with normal head circumference. Systemic examination revealed spleenohepatomegaly (spleen size 10 cm, liver size 6 cm), as shown in Figure 2.

The cardiovascular and central nervous system were normal. On fundus examination, there were signs suggestive of lipemia retinalis. A hemogram showed a white blood cell count of 13000 cells/μL, Hb of 6.1 g/dL, Hct of 18.8%, reticulocyte count of 6.7%, and platelet count of 260000 cells/μL, as shown in Table 1.

**Table 1:** Serial blood investigation reports of a child SGOT: Serum glutamic oxaloacetic transaminase; SGPT: Serum glutamic pyruvic transaminase; LDH: Lactate dehydrogenase; WBC: White blood cell

Parameters	On admission at 4months of life	Follow-up at 6 months	Follow-up at 8 months	Follow-up at 12 months
Hemoglobin (g/dL)	6.1	4.5	4.5	8
WBC (cells/μL)	13000	10000	11100	9500
Platelet (cells/μL)	2.6	1.2	1.8	2.2
Bilirubin (total/direct) (mg/dL)	2.2/0.36	2/0.7	1.84/0.57	2.2/0.6
SGOT/SGPT (U/L)	29/32	58/18	37/18	45/32
Triglyserides (mg/dL)	1197	907	281	280
Cholesterol (mg/dL)	117	100	117	124
Serum LDH (U/L)	3091.2	2400	700	432

The peripheral blood smear revealed polychromasia and nucleated erythrocytes. Direct and indirect Coombs’ tests were negative; the red blood cell membrane study was normal. The red blood cell enzyme study showed PK deficiency, with red cell PK activity at 4.7 IU/g, as shown in Table 2.

**Table 2:** Red blood cell enzyme assays MCF: Mean corpuscular fluorescence

Human red blood cell enzymes	Results	Normal range
Red cell pyruvate kinase	4.7 IU/g Hb	8-14 IU/g Hb
Glucose-6-phosphate dehydrogenase	6.27 IU/g Hb	4-13 IU/g Hb
Glucose phosphate isomerase	64.6 IU/g Hb	45-75 IU/g Hb
Red cell using eosin-5′-maleimide	925.0 MCF	900-1300 MCF

**Figure 3:**
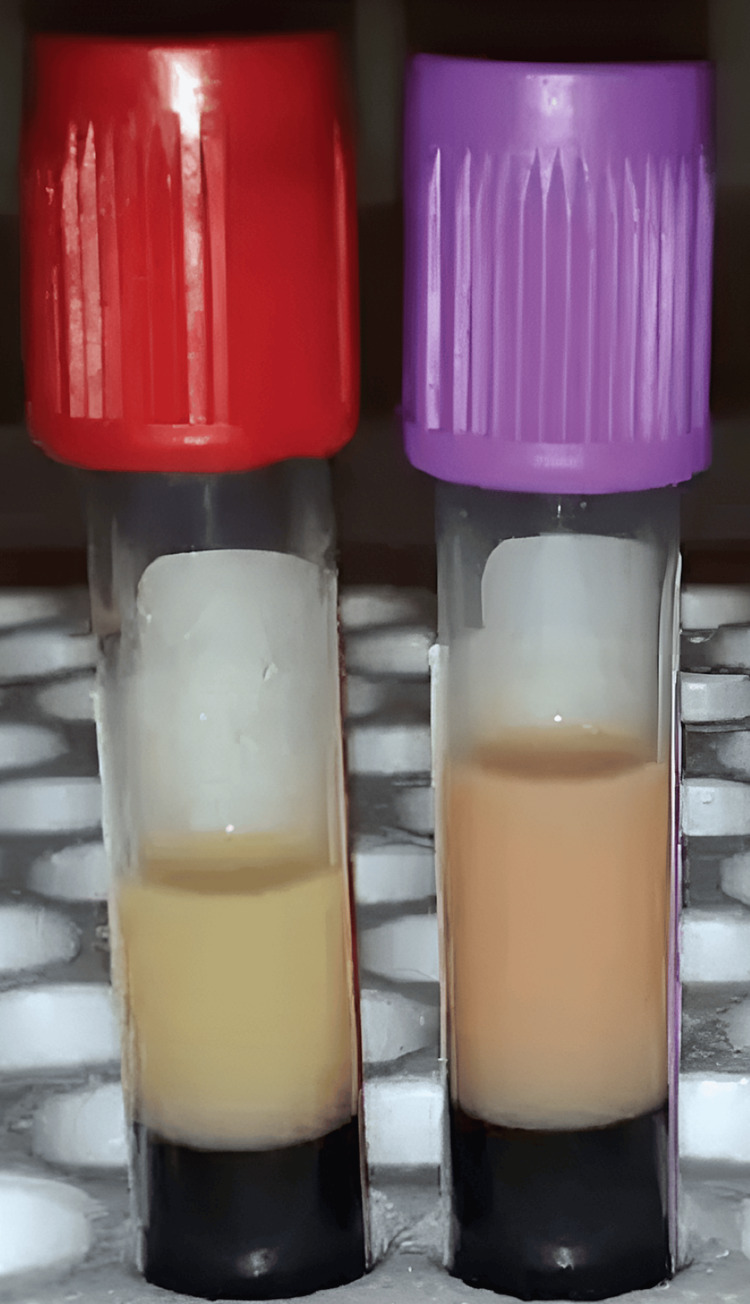
Milky blood sample

Serum lactate dehydrogenase (LDH) was 3091 U/L, suggesting hemolysis. Renal and liver function were normal, and high-performance liquid chromatography (HPLC) and bone marrow examination were normal. During blood sampling, we noticed a milky blood sample (Figure 3).

Hence, a lipid profile was obtained: serum triglyceride was 1197 mg/dL, and cholesterol was 119 mg/dL. Serum lipoprotein electrophoresis is suggestive of type IV hypertriglyceridemia, as shown in Table 3.

**Table 3:** Serum lipoprotein electrophoresis

Fractions	Results (mg/dL)	Reference range (%)
Alpha-lipoproteins	8	=<46.0
Pre-B-lipoproteins	41.9	=<29.0
Beta-lipoproteins	50.1	41-72

Ultrasonography showed splenohepatomegaly with normal liver texture. Ophthalmological examination revealed lipemia retinitis. Appropriate testing was used to rule out other causes of secondary hypertriglyceridemia, such as hypothyroidism, diabetes mellitus, and nephrotic syndrome. The mother had no laboratory evidence of hyperlipidemia. The father had died in a road traffic accident. A mutation study was advised to confirm the PK-LR gene mutation, but it was not done due to financial constraints. The child was given medium-chain fatty acid formula feed along with breastfeeding, blood transfusion, and folic acid supplements, and was advised to avoid salicylate groups of drugs. The lipid profile and complete blood count were repeated after two months, showing decreased serum triglycerides (564 mg/dL) and serum cholesterol (124 mg/dL) with low hemoglobin, so a blood transfusion was given. On examination, regression of spleen and liver size was noted. The child is receiving regular blood transfusions. Consequently, weight gain has been noted, and the child is developmentally normal.

## Discussion

The most common enzymopathy is red cell PK deficit, followed by glucose-6-phosphate dehydrogenase deficiency. It is caused by a mutation in a single gene, PK-LR [1]. Mutations in the PK-LR gene influence both the liver and red blood cell isozymes; however, only red cell glycolysis is severely compromised since mature erythrocytes are unable to replace damaged PK through fresh synthesis; only hepatocytes can. Hereditary disorders cause primary hypertriglyceridemia. Secondary hypertriglyceridemia is caused by high-fat diets, diabetes, obesity, hypothyroidism, and certain drugs (tamoxifen and estrogen). It is considered that epigenetic, environmental, and genetic factors contribute to the wide range of clinical manifestations. In this case report of red cell PK deficiency with severe hypertriglyceridemia, secondary hypertriglyceridemia is uncommon in individuals with chronic hemolytic anemia [3]. The known cases have been children with thalassemia major, which is associated with hypertriglyceridemia-thalassemia syndrome [5].

In other cases, serum triglyceride levels returned to normal after red cell transfusion therapy [5]. Malnutrition is also linked to hypertriglyceridemia in young children with thalassemia or PK deficiency [3]. Zieve syndrome (hyperlipidemia, jaundice, and transitory hemolytic anemia caused by alcohol abuse) demonstrates acquired PK deficiencies [6]. In a study of 36 patients with hemolysis, 27 had hypertriglyceridemia with hemolytic crisis; causes of hemolysis included microangiopathic hemolytic anemia, autoimmune hemolysis, infection, and glucose-6-phosphate dehydrogenase deficiency [4]. The case report of an eight-month-old female with thalassemia major and hypertriglyceridemia demonstrated clinical characteristics like classical thalassemia, except for a milky plasma [7]. This case shows the rare association of red cell PK deficiency with hypertriglyceridemia; it is usually resolved by dietary modification and blood transfusion therapy. The underlying pathogenesis is challenging to understand and requires molecular study.

## Conclusions

Early diagnosis and dietary modification can improve the prognosis and maintain a near-normal life for affected infants. Regular blood transfusions every month are required to maintain normal hemoglobin levels in PK deficiency disorder. Our case represents an unusual association of red cell PK deficiency with hypertriglyceridemia. This association could be coincidental or part of the same disease process. Molecular testing will be essential for a definitive diagnosis.
